# The Virtual Care Climate Questionnaire: Development and Validation of a Questionnaire Measuring Perceived Support for Autonomy in a Virtual Care Setting

**DOI:** 10.2196/jmir.6714

**Published:** 2017-05-08

**Authors:** Eline Suzanne Smit, Alexandra Lelia Dima, Stephanie Annette Maria Immerzeel, Bas van den Putte, Geoffrey Colin Williams

**Affiliations:** ^1^ Amsterdam School of Communication Research/ASCoR Department of Communication Science University of Amsterdam Amsterdam Netherlands; ^2^ Health Services and Performance Research (HESPER EA 7425) University Claude Bernard Lyon 1 Lyon France; ^3^ Trimbos Institute Netherlands Institute for Mental Health and Addiction Utrecht Netherlands; ^4^ Department of Clinical and Social Sciences in Psychology University of Rochester Rochester, NY United States; ^5^ Healthy Living Center, Center for Community Health Department of Medicine University of Rochester Rochester, NY United States

**Keywords:** questionnaire design, validation studies, psychometrics, personal autonomy, Internet, health behavior, health promotion, self-determination theory

## Abstract

**Background:**

Web-based health behavior change interventions may be more effective if they offer autonomy-supportive communication facilitating the internalization of motivation for health behavior change. Yet, at this moment no validated tools exist to assess user-perceived autonomy-support of such interventions.

**Objective:**

The aim of this study was to develop and validate the virtual climate care questionnaire (VCCQ), a measure of perceived autonomy-support in a virtual care setting.

**Methods:**

Items were developed based on existing questionnaires and expert consultation and were pretested among experts and target populations. The virtual climate care questionnaire was administered in relation to Web-based interventions aimed at reducing consumption of alcohol (Study 1; N=230) or cannabis (Study 2; N=228). Item properties, structural validity, and reliability were examined with item-response and classical test theory methods, and convergent and divergent validity via correlations with relevant concepts.

**Results:**

In Study 1, 20 of 23 items formed a one-dimensional scale (alpha=.97; omega=.97; *H*=.66; mean 4.9 [SD 1.0]; range 1-7) that met the assumptions of monotonicity and invariant item ordering. In Study 2, 16 items fitted these criteria (alpha=.92; *H*=.45; omega=.93; mean 4.2 [SD 1.1]; range 1-7). Only 15 items remained in the questionnaire in both studies, thus we proceeded to the analyses of the questionnaire’s reliability and construct validity with a 15-item version of the virtual climate care questionnaire. Convergent validity of the resulting 15-item virtual climate care questionnaire was confirmed by positive associations with autonomous motivation (Study 1: *r*=.66, *P<*.001; Study 2: *r*=.37, *P<*.001) and perceived competence for reducing alcohol intake (Study 1: *r*=.52, *P<*.001). Divergent validity could only be confirmed by the nonsignificant association with perceived competence for learning (Study 2: *r*=.05, *P=*.48).

**Conclusions:**

The virtual climate care questionnaire accurately assessed participants’ perceived autonomy-support offered by two Web-based health behavior change interventions. Overall, the scale showed the expected properties and relationships with relevant concepts, and the studies presented suggest this first version of the virtual climate care questionnaire to be reasonably valid and reliable. As a result, the current version may cautiously be used in future research and practice to measure perceived support for autonomy within a virtual care climate. Future research efforts are required that focus on further investigating the virtual climate care questionnaire's divergent validity, on determining the virtual climate care questionnaire’s validity and reliability when used in the context of Web-based interventions aimed at improving nonaddictive or other health behaviors, and on developing and validating a short form virtual climate care questionnaire.

## Introduction

Unhealthy lifestyle behaviors—for example, smoking tobacco or cannabis, consuming too much alcohol, overeating calories or not eating a sufficient amount of fruit and vegetables, or being insufficiently physically active—are a major cause of chronic illnesses like cancer, diabetes, and cardiovascular diseases [1]. They have a detrimental effect on quality of life, decrease work productivity, and put an enormous, preventable strain on health care [2]. Developing effective interventions to promote healthy lifestyle behaviors that prevent or delay these diseases, as well as making these interventions available on a large scale and to a great variety of people, is thus important.

To improve one’s lifestyle, self-determination theory (SDT) [3] proposes that an autonomous form of motivation and perceiving competence for changing are imperative. When it concerns the improvement of health-related behaviors like those mentioned before, people are assumed to perceive themselves to be autonomous in their motivation to change when the behavior is accompanied by an experience of psychological freedom of choice. Supportive of this theoretical assumption, earlier research has found autonomous motivation to be an important predictor of health behavior change, its maintenance, and subsequent positive health outcomes [4]. According to SDT, someone’s autonomous motivation can be increased by providing **s**upport for autonomy. The concept of autonomy-support was first of all applied to a face-to-face context where one person, such as a health professional, interacts with another person, such as a patient, and tools have been developed to measure this concept in face-to-face settings. When provided by a health care professional, such as a general practitioner or lifestyle counselor, support for autonomy involves strategies like eliciting and acknowledging a person’s perspective, providing a clear rationale for change, offering choice, and using noncontrolling language [5,6]. Perceived support for autonomy from a health care professional is measured through instruments like the health care climate questionnaire (HCCQ) [7]. Similarly, perceived autonomy-support from a physical education teacher can be assessed by the perceived autonomy support scale for exercise settings (PASSES) [8].

Nowadays, interventions aimed at a healthy lifestyle are increasingly delivered via the Internet [9,10], creating a virtual instead of face-to-face care climate. Web-based health behavior change interventions can successfully promote a healthy lifestyle [11] and have several advantages over and above face-to-face interventions; they are highly accessible, participants can use them at any convenient time, and many people can be reached at minimal cost [9]. It can be assumed to be equally important to provide support for autonomy within a virtual care climate in order to increase people’s autonomous motivation for initiating and maintaining health behavior change. However, instead of human interaction, Web-based interventions deliver this support in different formats and rather make use of computer-human interaction. For interventions that make use of virtual health care providers, like a virtual clinician [12] or computerized personal trainer [13], the interaction between the virtual health care provider and receiver of the intervention might resemble the interaction between two humans. Consequently, only slight adjustments to questionnaires like the HCCQ and PASSES might be sufficient. There is, however, an abundance of Web-based interventions that do not involve virtual care providers, but in which autonomy-support is integrated in the structure of the Web-based tool (for examples of such interventions, see [14-16]). In this context, the operationalization of the concept of perceived autonomy-support would need to be reconsidered to a larger extent and available measurement instruments would need to be adapted to this new context. To illustrate, one of the items from the HCCQ reads, “My physician listens to how I would like to do things;” whereas a physician or other person is indeed able to listen, a Web-based intervention where no virtual health care provider is involved does not possess this ability. As a consequence, the item would not be applicable.

Although substantial evidence is available for the positive effects of perceived autonomy-support within the face-to-face setting [4,6], as well as some evidence for these effects when it concerns virtual health care providers [12,13], no such evidence exists for the role of autonomy-support in virtual care settings without virtual health care providers involved. To develop an evidence base for this growing field of Web-based interventions, an instrument is needed with adequate measurement properties specifically for assessing perceived support for autonomy in such settings. Therefore, the objective of this study was to develop and validate the virtual care climate questionnaire (VCCQ): the first measurement instrument of perceived support for autonomy in virtual care settings. By developing the VCCQ, this study aimed to fulfill the need for such tools and thus enable further research into how Web-based health behavior change interventions can successfully support autonomy, increase autonomous motivation, and ultimately promote a healthy lifestyle.

## Methods

The VCCQ was constructed in three steps: item development, pretesting, and psychometric validation.

### Item Development

The items of the VCCQ were developed primarily based on the HCCQ [7]. The 15 items of the HCCQ were adapted to measure perceived autonomy-support in a virtual instead of face-to-face care setting. The adaptation of the items consisted of rewording items from a setting in which there is direct contact with a physician (eg, “My physician answers my questions fully and carefully”) to a setting in which the respondent interacts with a Web-based intervention (eg, “< *name intervention*> answers my questions fully and carefully”). Additionally, the target behavior was added when applicable to increase the specificity of the items (eg, “I feel that <name intervention> provides me with effective possibilities to <target behavior>”). The name of the new questionnaire, that is, virtual care climate questionnaire, aims to recognize the basis of most of its items, that is, the health care climate questionnaire. Yet, to ensure that a wide variety of autonomy-supportive strategies were represented in the VCCQ, four additional items were included based on the PASSES [8] (items 16 and 17) and a discussion with experts on motivation and Web-based health behavior change interventions (items 18 and 19).

Similar as in the HCCQ, a 7-point response scale was used with *totally disagree* (1) and *totally agree* (7) as endpoints. All items and possible responses were translated from English to Dutch.

### Pretesting

To investigate the face validity of the VCCQ, a pretest was conducted among experts. Moreover, a pretest was conducted among the target population in order to identify improving alterations to the questionnaire.

#### Sample and Procedure

Pretests were conducted in the context of a Web-based, computer-tailored intervention aimed at reducing alcohol intake (*Drinktest* [15]) and took place among five experts as well as five Dutch adults who (occasionally) drink alcohol, with varying age and socioeconomic status.

The experts were identified through the first author’s professional network and were considered experts when they had a track record in the field of motivation, health behavior, or Web-based health behavior change interventions. Experts were asked whether they thought the 19-item VCCQ measured the same concept as the HCCQ, whether items were properly reworded to fit a virtual care setting, and whether they thought the VCCQ was a comprehensive instrument to measure perceived autonomy-support in a virtual care setting. Furthermore, experts were asked to indicate any perceived ambiguities in wording and whether they felt any aspects of autonomy-support were not covered. Experts also gave feedback on the translation of the items from English to Dutch.

The Dutch adults were invited to complete the VCCQ after they had visited and consulted the Web-based intervention Drinktest, and reflected on whether the questions and instructions in the VCCQ were clearly formulated and whether they thought the questionnaire had an acceptable length *.* During their participation, they were asked to take notes, which were discussed with them afterwards.

#### Pretest Results

Most experts indicated that, generally due to the rewording of the HCCQ-items to a virtual care setting, the VCCQ items did not always measure the underlying concept of perceived support for autonomy. To ensure the VCCQ truly measured perceived autonomy-support in a virtual care setting, experts suggested to primarily use the content of the HCCQ-items as a basis and reformulate items when necessary, instead of a literal translation and rewording to the virtual context. Furthermore, experts indicated the VCCQ to be reasonably comprehensive. Yet, to ensure the VCCQ included a wide range of autonomy-supportive strategies, four additional items were included based on their feedback (items 20-23). Moreover, based on both expert input and feedback from the participating Dutch adults, the wording of 14 VCCQ-items was simplified to guarantee comprehensibility by the target group. This resulted in the final 23-item questionnaire presented in Table 1.

**Table 1:** The 23 items of the initial VCCQ (virtual care climate questionnaire) with their description and source.

No.	Item	Item description	Source
1	VCCQ_choice^a^	I feel that <name intervention> has provided me with choices and options	HCCQ^b^
2	VCCQ_understood	I felt understood by <name intervention>	HCCQ
3	VCCQ_honest	I am able to be honest and open when completing questions about <target behavior> on <name intervention>	HCCQ
4	VCCQ_confidence^a^	<name intervention> conveys confidence in my ability to <target behavior>	HCCQ
5	VCCQ_judgment^a^	I feel that <name intervention> does not judge me	HCCQ
6	VCCQ_knowledge	Because of <name intervention> I really understand what I need to do to <target behavior>	HCCQ
7	VCCQ_answers^a^	<name intervention> encourages me to search for answers to the questions that I have	HCCQ
8	VCCQ_trust^a^	I feel a lot of trust in <name intervention>	HCCQ
9	VCCQ_questions^a^	<name intervention> answers my questions fully and carefully	HCCQ
10	VCCQ_input^a^	<name intervention> allows me to provide input on how I would like to do things	HCCQ
11	VCCQ_emotions^a^	<name intervention> takes into account my emotions in the advice given	HCCQ
12	VCCQ_care^a^	I feel that <name intervention> cares about me as a person	HCCQ
13	VCCQ_communication	I don’t feel very good about the way <name intervention> communicates with me	HCCQ
14	VCCQ_see^a^	<name intervention> tries to incorporate how I see things in the advice given	HCCQ
15	VCCQ_feelings	I feel that <name intervention> asks enough questions about my feelings	HCCQ
16	VCCQ_stimulant^a^	<name intervention> encourages me to <target behavior>	PASSES^c^
17	VCCQ_feedback	<name intervention> provides me with positive feedback when I do something to <target behavior>	PASSES
18	VCCQ_steering	I feel the advice of <name intervention> is directive	Experts
19	VCCQ_effective^a^	I feel that <name intervention> provides me with effective possibilities to <target behavior>	Experts
20	VCCQ_way^a^	<name intervention> gives me the feeling that I can choose a way to <target behavior> myself	Experts
21	VCCQ_idea^a^	<name intervention> explains to me why it would be a good idea to <target behavior>	Experts
22	VCCQ_use^a^	<name intervention> asks me the right questions about <target behavior>	Experts
23	VCCQ_must	I feel that <name intervention> is telling me what to do about <target behavior> without my having a say	Experts

^a^Items were included in the final VCCQ (virtual care climate questionnaire) based on the psychometric validation; the Dutch version of the final VCCQ is available upon request.

^b^HCCQ: health care climate questionnaire.

^c^PASSES: perceived autonomy support scale for exercise settings.

### Psychometric Validation

To validate the VCCQ psychometrically, two studies were conducted. Study 1 was conducted in the context of a Web-based, computer-tailored intervention to reduce alcohol intake (Drinktest [15,17]) among Dutch adults who drink alcohol (occasionally). Study 2 was conducted in the context of a Web-based, computer-tailored intervention to reduce cannabis intake (*Weed-check* [18]) among a sample of Dutch students.

#### Sample and Procedure

##### Study 1

The sample consisted of 230 Dutch adults who drink alcohol (occasionally). Participants were recruited via the ISO-certified (International Organization for Standardization, ISO) research panel *PanelClix* [19] and, after providing informed consent, completed the study through the Web. Before filling out the VCCQ, participants visited and consulted Drinktest [17], a Dutch, Web-based, computer-tailored intervention to reduce alcohol intake [15]. When consulting this intervention, participants answered a variety of questions concerning their current alcohol consumption as well as their cognitions related to this behavior, based on which they received feedback that is tailored to their personal situation and beliefs. This feedback was presented to participants on their computer screen, and participants were also to send this feedback to an email address of their choice as well as to print it. To ensure that participants made proper use of the Web-based intervention, they were instructed to imagine they wanted to reduce their alcohol intake and that they used the Web-based intervention to this end. For participants to be able to imagine wanting to reduce their alcohol intake, an inclusion criterion was set based on participants’ alcohol intake. Participants who indicated never to have drunk alcoholic beverages before or those who had not drunk any alcoholic beverages in the past 12 months were excluded from participation—as were respondents who did not complete the survey entirely, had missing data on key variables, did not engage with the intervention, or participated for too long in the survey (ie, had z-scores >3 for participation time). After visiting and consulting the Web-based intervention, they were informed that they would be asked for their opinions about—and personal experiences with—this intervention in a subsequent questionnaire (ie, the VCCQ) and to answer several related questions regarding their motivation and perceived competence for changing. Participants were rewarded for their approximately 15-minute participation by means of 100 *Clix*, the usual incentive used by PanelClix, worth about €1.25.

##### Study 2

The sample consisted of 228 Dutch students using cannabis. Participants were recruited via the student participation website of the faculty of Social and Behavioral Sciences of the University of Amsterdam and, after providing informed consent, completed the study through the Web. As part of the survey, participants visited and consulted Weed-check [18], a Dutch, Web-based, computer-tailored intervention to reduce cannabis consumption. Similar as in the intervention used for Study 1, as part of their consultation of this intervention participants answered questions concerning their cannabis consumption and their cognitions associated with cannabis consumption, based on which they received feedback that was tailored to their personal situation and beliefs. Again, feedback was visible on the participants’ computer screen, and options were provided to send the feedback to an email address and to print it. To ensure that participants made proper use of the Web-based intervention, they were instructed to imagine they wanted to have an overview of their cannabis usage and that they used the Web-based intervention to this end. In order for participants to be able to imagine wanting an overview of their cannabis consumption, an inclusion criterion was set based on participants’ cannabis usage. Participants who indicated never to have used cannabis before or those who had used cannabis but only for medical reasons were excluded—as were respondents who did not complete the survey entirely, participated twice, indicated to have no understanding of the intervention, participated for too long in the survey, or had a too high number of neutral answers (ie, had z-scores >3 for participation time and the number of neutral answers, respectively). After visiting and consulting the Web-based intervention, participants were informed that they would be asked for their opinions about—and personal experiences with—this intervention and to answer several related questions regarding their motivation and perceived competence for changing. As a reward for their approximately 20-minute participation, students could choose their own incentive (ie, 0.5 research credits or € 2.50).

#### Measures

Both studies assessed several common and study-specific background variables, autonomous motivation to change, and perceived competence concepts, as detailed below.

##### Background Variables

Participants’ age and gender were assessed via single items. Moreover, in Study 1 several characteristics of alcohol consumption were measured: the total number of weekdays consuming alcoholic beverages, average number of alcoholic beverages consumed during a typical weekday, total number of weekend days consuming alcoholic beverages, average number of alcoholic beverages consumed during a typical weekend day, and the total number of times consuming more than four alcoholic beverages in a day within the last 6 months. In Study 2, four characteristics of cannabis consumption were measured: number of times consuming cannabis in the last 12 months, cannabis use in the last 30 days, number of times consuming cannabis in the last 30 days, total number of joints on a typical day.

##### Autonomous Motivation

The degree to which one’s motivation to change is autonomous (vs controlled) was measured by the treatment self-regulation questionnaire (TSRQ) [20], consisting of 16 items relating to autonomous or controlled motivation, or amotivation. The autonomous motivation subscale (6 items) showed good internal consistency (Study 1: Cronbach alpha=.95 and omega=.93; Study 2: Cronbach alpha=.91 and omega=.91), as did the controlled motivation subscale (7 items; Study 1: Cronbach alpha=.93 and omega=.93; Study 2: Cronbach alpha =.92 and omega=.92).

##### Perceived Competence for Changing

Participants’ feelings of competence to change were measured by the perceived competence scale (PCS) [21] for reducing alcohol intake (Study 1) and cannabis intake (Study 2), respectively. The PCS asked participants the extent to which they agreed with the four statements, such as “I feel confident in my ability to reduce my alcohol intake” (Study 1), or “I am capable to reduce my cannabis consumption” (Study 2). All items were measured on a scale ranging from *strongly disagree* (1) to *strongly agree* (7), and the scale proved to be reliable (Study 1: Cronbach alpha=.95 and omega=.95; Study 2: Cronbach alpha=.93 and omega=.93). For divergent validity analysis purposes, in Study 2 perceived competence for learning was also assessed using the PCS [22] (Cronbach alpha=.94 and omega=.94).

#### Analyses

All statistical analyses were conducted using R statistical package [23]. Item selection followed several steps following the criteria described below.

##### Item Descriptives and Inter-Item Correlations

The first step was to investigate the distribution of responses to VCCQ items via descriptive statistics and examine the strength of association between items based on Spearman correlations. Items were flagged if they showed inadequate distributions (eg, <10 responses per response option [24]) or low associations with other items (eg, nonsignificant correlations with other items), but were kept in further analyses for detailed diagnosis.

##### Nonparametric Item Response Theory Analyses

To examine the VCCQ’s structural validity taking into account variations in item difficulty [25], nonparametric item response theory (NIRT) analyses (Mokken scale analysis, MSA) were conducted using the *mokken* package in R [26]. MSA examines whether an item set orders respondents accurately on a continuum representing a single latent trait (ie, in this study perceived autonomy-support). According to MSA, items can be considered a scale (fit the monotone homogeneity model) if three conditions are met: unidimensionality, monotonicity, and local independence (LI). If the items also meet a fourth criterion, invariant item ordering (IIO), the scale can be used for group comparison [25,27].

Unidimensionality entails that items can be located on a single latent continuum in terms of probabilities of obtaining high scores [25]. It was examined by assessing coefficients of homogeneity (*H*; ranging from 0 to 1, from no association to perfect association considering item distributions) and via an automated item selection procedure (*aisp*) algorithm [26]. Loevinger’s scalability coefficients estimated item and scale homogeneity of the items (*H*_i_ coefficients and *H* coefficients) [28]. The *aisp* analysis performs an exploration of the scale unidimensionality at increasing levels of homogeneity [28]. Monotonicity implies that the probability of obtaining high scores on an item does not decrease as latent trait scores increase; *crit* values >40 are considered as violating monotonicity requirements [29] and then the recommended strategy is to remove the item with the most serious (highest) violation and rerun the analysis. LI means that associations between items are explained only by their relationship with the latent trait (perceived autonomy-support) [26]. At the moment, LI is still under research as a new MSA test [30]; until very recently, no tests were even available to test this assumption. Therefore, in this study no exclusion decisions were made based on LI results. If the items also show IIO, the scale represents a “person-free” item hierarchy in terms of their difficulty, that is, the order of items remains the same at different levels of the latent dimension [31]. LI and IIO are tested via dedicated functions that flag items that violate these criteria to be considered for exclusion. For monotonicity and IIO, minimum group size was set at 50; LI was examined with default parameter values.

##### Confirmatory Factor Analysis and Reliability

After selecting an item set fitting the MSA criterion, a confirmatory factor analysis (CFA) was conducted using the function *cfa* in the *lavaan* R package [32] to examine whether the unidimensional structure is supported by this alternative analysis, and thus allows comparisons with prior literature on autonomy-support scales; default parameter values were used (eg, maximum likelihood estimator, covariance matrix). Model diagnostics, parameter estimates, and goodness-of-fit indices were examined. Model fit was judged against the following criteria: Tucker-Lewis index (TLI) and Comparative Fit Index (CFI) >0.95; root mean square error of approximation (RMSEA) <0.06; and chi-square *P* value >.05 [33,34]. This analysis was followed by assessing the internal consistency of the final scale using Cronbach alpha and omega [35].

##### Construct Validity

The VCCQ’s construct validity was determined by examining its convergent and divergent validity, based on expectations based on theory [3] and evidence from earlier research [8]. In terms of convergent validity, the VCCQ was expected to positively correlate with the autonomous motivation subscale of the TSRQ as well as with perceived competence for changing (ie, for reducing one’s alcohol intake or cannabis consumption). In terms of divergent validity, the VCCQ was expected to show nonsignificant or negative correlations with the controlled motivation subscale of the TSRQ and with the nonhealth-related concept of perceived competence for learning (Study 2 only). Both studies had 90% power to detect bivariate correlations *r*=.21 and 80% power for *r*=.18, at alpha=.05.

## Results

### Samples

Sample characteristics of respondents participating in Study 1 are presented in Table 2. For Study 2, sample characteristics are presented in Table 3. For both studies, the flow of respondents is presented Multimedia Appendix 1.

**Table 2:** Demographic characteristics of respondents participating in Study 1 (N=230).

Variable	Response categories	n (%)
Gender	Female	110 (47.8)
	Male	120 (52.2)
Age, mean (SD)		46.09 (15.29)
Number of weekdays alcoholic beverages are used	4	49 (21.3)
	3	16 (7.0)
	2	40 (17.4)
	1	46 (20)
	Less than 1	42 (18.3)
	I never drink alcoholic beverages during weekdays	37 (16.1)
How many glasses do you usually drink during a weekday?	11 or more	3 (1.3)
	7-10	4 (1.7)
	6	9 (3.9)
	5	7 (3.0)
	4	11 (4.8)
	3	21 (9.1)
	2	79 (34.3)
	1	59 (25.7)
Number of weekend days alcoholic beverages are used	3	78 (33.9)
	2	68 (29.6)
	1	55 (23.9)
	Less than 1	29 (12.6)
How many glasses do you usually drink during a weekend day?	11 or more	8 (3.5)
	7-10	19 (8.3)
	6	10 (4.3)
	5	13 (5.7)
	4	31 (13.5)
	3	45 (19.6)
	2	69 (30.0)
	1	35 (15.2)
How often have you drunk four or more glasses in a day within the last 6 months?	Every day	17 (7.4)
	5-6 times per week	3 (1.3)
	3-4 times per week	18 (7.8)
	1-2 times per week	31 (13.5)
	1-3 times per month	34 (14.8)
	3-5 times per 6 months	25 (10.9)
	1-2 times per 6 months	43 (18.7)
	Never	59 (25.7)

**Table 3:** Demographics characteristics of respondents participating in Study 2 (N=228).

Variable	Response categories	n (%) or mean (SD)
Gender	Female	157 (68.9)
	Male	71 (31.1)
Age in years, mean (SD)		21.44 (2.30)
How often have you used cannabis in the last 12 months? n (%)	1 time	25 (11.0)
	2 times	45 (19.7)
	3 times	36 (15.8)
	4 times	24 (10.5)
	5 times or more	98 (43.0)
Used cannabis in the last 30 days, n (%)	Yes	118 (51.8)
	No	110 (48.2)
How often have you used cannabis in the last 30 days? n (%)	Daily	14 (6.1)
	More times per week	10 (4.4)
	At least 1 time per week	21 (9.2)
	Less than 1 time per week	73 (32.0)
Number of joints used on a typical day, mean (SD)		1.2 (0.88)

### Item Descriptives and Inter-Item Correlations

In Study 1, participants’ average scores on the 23 VCCQ-items ranged between 3.68 (SD 1.66) and 5.59 (SD 1.29) (Table 4). Scores covered the entire range on the scale from 1 to 7. All items were skewed toward agreement that the virtual care climate is autonomy-supportive, though varied in the extent to which agreement was expressed (Multimedia Appendix 2). Only two items had ≥10 answers in each of the two response categories at the lower end of the scale. Inter-item correlations ranged from −.11 to .79 (*P*<.001) (Multimedia Appendix 3) *.* Two negatively worded items (13 and 23) showed nonsignificant associations with other items.

In Study 2, participants’ average scores on the 23 VCCQ-items ranged between 3.70 (SD 1.61) and 6.01 (SD 1.12) (Table 4). As in Study 1, scores covered the entire range of the scale. When compared with Study 1, items were less skewed toward agreement that the virtual care climate is autonomy-supportive; three items had <10 answers for the response category at the lower extreme and 14 items had <10 answers for the highest response category (Multimedia Appendix 2). Inter-item correlations (Spearman) ranged from −.22 (*P*<.001) to .67 (*P*<.001) (Multimedia Appendix 3) *.* The same two negatively worded items (13 and 23) as in Study 1 as well as item 18 and item 3 showed weak or no associations with other items.

Nonetheless, all items in both studies were included in the next steps for a more detailed diagnosis.

**Table 4:** Descriptive statistics for the 23 VCCQ (virtual care climate questionnaire) items in Study 1 (N=230) and Study 2 (N=228).

No.	VCCQ (virtual care climate questionnaire) items	Study 1		Study 2	
		Mean (SD)	Skew	Mean (SD)	Skew
1	VCCQ_choice	5.06 (1.32)	−0.34	4.46 (1.78)	−0.49
2	VCCQ_understood	4.97 (1.29)	−0.24	3.79 (1.69)	−0.13
3	VCCQ_honest	5.59 (1.29)	−0.75	6.01 (1.12)	−1.49
4	VCCQ_confidence	4.70 (1.32)	−0.19	4.01 (1.49)	−0.35
5	VCCQ_judgment	5.19 (1.37)	−0.54	4.58 (1.84)	−0.43
6	VCCQ_knowledge	4.78 (1.33)	−0.17	3.73 (1.51)	−0.10
7	VCCQ_answers	4.73 (1.34)	−0.13	3.70 (1.61)	−0.04
8	VCCQ_trust	4.96 (1.27)	−0.21	4.09 (1.58)	−0.30
9	VCCQ_questions	4.91 (1.24)	−0.11	4.21 (1.51)	−0.38
10	VCCQ_input	4.72 (1.21)	0.07	3.75 (1.50)	−0.11
11	VCCQ_emotions	4.96 (1.32)	0.02	4.03 (1.56)	−0.29
12	VCCQ_care	4.88 (1.32)	−0.21	3.86 (1.68)	−0.29
13	VCCQ_communication	3.68 (1.66)	0.05	3.85 (1.66)	0.09
14	VCCQ_see	4.70 (1.14)	0.24	4.10 (1.51)	−0.43
15	VCCQ_feelings	4.53 (1.33)	0.04	4.14 (1.47)	−0.24
16	VCCQ_stimulant	4.77 (1.33)	−0.13	4.19 (1.78)	−0.25
17	VCCQ_feedback	4.84 (1.22)	−0.01	4.28 (1.33)	−0.57
18	VCCQ_steering	4.59 (1.24)	−0.03	4.92 (1.42)	−0.68
19	VCCQ_effective	4.79 (1.18)	−0.14	4.11 (1.50)	−0.39
20	VCCQ_way	4.90 (1.21)	−0.20	4.28 (1.42)	−0.51
21	VCCQ_idea	5.08 (1.24)	−0.17	5.12 (1.45)	−0.94
22	VCCQ_use	5.10 (1.21)	−0.22	4.51 (1.59)	−0.62
23	VCCQ_must	4.19 (1.46)	−0.20	4.25 (1.65)	−0.05

### Nonparametric Item Response Theory Analyses

In Study 1, items 13 and 23 violated the assumption of unidimensionality (ie, item *H*<.30; Table 5); therefore, these items were excluded from further analyses. Table 5 therefore shows results for the resulting 21-item unidimensionality analysis. All items and the total scale had scores >.30 (*H=*.660, standard error, SE=0.029), hence the 21-item VCCQ scale could be considered unidimensional. In other words, its items measured a single underlying concept, as intended. No significant violations of monotonicity were found for the 21-item VCCQ (ie, all *crit* values <.40). Violations of IIO were only found for item 6; therefore, this item was excluded. All 20 remaining items fitted the IIO criterion.

In Study 2, items 13, 23, 18, and 3 were found to have item *H<*.30 (Table 5) and were therefore excluded. Table 5 shows the results of the unidimensionality analysis of the remaining 19 items. All item *H* s and the scale *H* were >.30 (*H=.* 445, SE=0.030). The results confirmed that the 19-item VCCQ scale could be considered unidimensional. No significant violations of monotonicity were found (ie, all *crit* values <.40) Violations of IIO were found for items 15, 17, and 2, which were therefore excluded, resulting in a final 16-item VCCQ.

With regard to LI, in Study 1 items 11 and 14 and in Study 2 items 16, 20, and 21 were flagged as possibly violating LI – whereas these items were retained in the questionnaire in this study as LI is still under construction, these items may be considered for exclusion when developing a VCCQ short-form.

**Table 5:** Loevinger’s scalability coefficients (*H*) for the 23-item and 21-item VCCQ (virtual care climate questionnaire) in Study 1 (N=230) and the 23-item and 19-item VCCQ in Study 2 (N=228).

No.	Description	Study 1		Study 2	
		23-item	21-item	23-item	19-item
		H_i_(SE)^a^	H_i_(SE)	H_i_(SE)	H_i_(SE)
1	VCCQ_choice	0.563 (0.043)	0.627 (0.045)	0.333 (0.037)	0.409 (0.042)^b^
2	VCCQ_understood	0.599 (0.034)	0.665 (0.035)	0.429 (0.028)	0.519 (0.031)^b^
3	VCCQ_honest	0.493 (0.047)	0.555 (0.050)	0.096 (0.045)	Excluded
4	VCCQ_confidence	0.604 (0.033)	0.659 (0.035)	0.366 (0.034)	0.431 (0.038)
5	VCCQ_judgment	0.596 (0.036)	0.667 (0.037)	0.345 (0.035)	0.434 (0.039)^b^
6	VCCQ_knowledge	0.620 (0.032)	0.688 (0.032)^b^	0.394 (0.035)	0.463 (0.040)
7	VCCQ_answers	0.607 (0.031)	0.671 (0.032)	0.402 (0.033)	0.462 (0.037)
8	VCCQ_trust	0.615 (0.035)	0.686 (0.035)	0.402 (0.031)	0.479 (0.035)
9	VCCQ_questions	0.643 (0.029)	0.713 (0.029)	0.412 (0.032)	0.488 (0.035)
10	VCCQ_input	0.622 (0.032)	0.681 (0.033)	0.392 (0.036)	0.471 (0.040)
11	VCCQ_emotions	0.594 (0.034)	0.662 (0.034)	0.412 (0.032)	0.492 (0.035)
12	VCCQ_care	0.638 (0.028)	0.707 (0.028)	0.399 (0.033)	0.480 (0.037)
13	VCCQ_communication	−0.011 (0.075)	Excluded	−0.043 (0.053)	Excluded
14	VCCQ_see	0.604 (0.034)	0.651 (0.037)	0.398 (0.034)	0.478 (0.038)
15	VCCQ_feelings	0.560 (0.036)	0.610 (0.038)	0.270 (0.039)	0.320 (0.045)
16	VCCQ_stimulant	0.596 (0.037)	0.649 (0.040)	0.321 (0.039)	0.381 (0.045)
17	VCCQ_feedback	0.626 (0.035)	0.685 (0.036)	0.360 (0.040)	0.406 (0.046)^b^
18	VCCQ_steering	0.511 (0.054)	0.548 (0.059)	0.151 (0.051)	Excluded
19	VCCQ_effective	0.638 (0.032)	0.691 (0.035)	0.406 (0.035)	0.473 (0.040)
20	VCCQ_way	0.654 (0.028)	0.719 (0.029)	0.402 (0.036)	0.486 (0.039)
21	VCCQ_idea	0.576 (0.040)	0.648 (0.041)	0.318 (0.041)	0.363 (0.045)
22	VCCQ_use	0.610 (0.042)	0.678 (0.045)	0.353 (0.038)	0.417 (0.043)
23	VCCQ_must	0.195 (0.077)	Excluded	−0.003 (0.054)	Excluded
	Scale	H (SE)	H (SE)	H (SE)	H (SE)
	VCCQ	0.547 (0.029)	0.660 (0.029)	0.321 (0.027)	0.445 (0.030)

^a^SE: standard error.

^b^Item was excluded based on violation of the IIO assumption.

Only 15 items remained in the VCCQ in both Study 1 and Study 2. For research as well as clinical purposes it is most desirable to keep the respondent burden of questionnaire completion as low as possible, and use the same items across interventions aimed at different behaviors. Therefore, we proceeded to the analyses of the questionnaire’s reliability and construct validity with a 15-item version of the VCCQ. The remaining 15 items maintained the breadth of the theoretical concept. That is, an item was only excluded when its content showed overlap with other items (six instances), it was excluded based on item response theory (IRT) results in both studies (one instance), or when the item was retrospectively deemed inappropriate in the context of Web-based, computer-tailored interventions (one instance). Remaining items are presented in bold font in Table 1. Item step response functions of the 15 remaining items are presented in Multimedia Appendix 4.

### Confirmatory Factor Analysis and Reliability

The CFA with data from Study 1 showed that the CFI of the one-factor model was 0.897, the TLI was 0.879, and the RMSEA was 0.128 (95% CI 0.115-0.140). In Study 2, results were similar: CFI=0.902; TLI=0.885; RMSEA=0.087 (95% CI 0.074-0.100). Chi-square tests were significant for both models (χ^2^_90_=426.6 and 244.8, *P<*.001). The scale showed good internal consistency both in Study 1 (Cronbach alpha=.97 and omega=.97, *H*=.66, mean 4.9 [SD 1.0]) and Study 2 (Cronbach alpha=.92 and omega=.92, *H*=.66, mean 4.9 [SD 1.0]). CFA diagrams are presented in Multimedia Appendix 5.

### Construct Validity

#### Convergent Validity

In Study 1, the correlation between the final 15-item VCCQ and the autonomous motivation subscale of the TSRQ was strong and positive (*r*=.66, *P<*.001). That is, the extent to which participants perceived Drinktest to be autonomy-supportive—as measured by the VCCQ—was significantly and positively correlated with participants’ autonomous motivation to reduce their alcohol intake. Furthermore, the correlation between the VCCQ and PCS for reducing alcohol intake was also strong and positive (*r*=.52, *P<*.001). The extent to which participants perceived Drinktest to be autonomy-supportive was significantly and positively correlated to participants’ perceived competence for reducing their alcohol intake. These findings support the hypothesis of positive relationships between perceived autonomy-support in a virtual care setting and both autonomous motivations and perceived competence for reducing alcohol intake, indicating the convergent validity of the VCCQ.

In Study 2, the correlation between the final 15-item VCCQ and the autonomous motivation subscale of the TSRQ was moderately strong and positive (*r*=.37, *P<*.001), whereas the correlation between the VCCQ and the PCS for cannabis reduction was weak and not significant (*r*=.01, *P=*.12).

#### Divergent Validity

In line with expectations, results from Study 1 showed that the correlation between the VCCQ and the controlled motivation subscale of the TSRQ was moderate, though contrary to expectations this correlation was positive (*r=*.29, *P<*.001). The extent to which participants perceived Drinktest to be autonomy-supportive was significantly and positively correlated with participants’ controlled motivations to reduce their alcohol intake—though the correlation was much weaker compared with the correlation between the VCCQ and autonomous motivation. Consequently, some support was found for the hypothesized divergent validity of the VCCQ.

In Study 2, similar results were found: the correlation between the VCCQ and the controlled motivation subscale of the TSRQ was moderately strong and positive (*r*=.37, *P<*.001)—this correlation had a similar strength as the correlation between the VCCQ and autonomous motivation. In line with expectations, the association between the VCCQ and the PCS for learning was weak and not significant (*r*=.05, *P=*.48), supporting the divergent validity of the VCCQ. Together, however, the results concerning the scale’s divergent validity in this study were mixed.

Table 6 summarizes results for convergent and divergent validity in both studies.

**Table 6:** Pearson correlations between the virtual care climate questionnaire, the treatment self-regulation questionnaire autonomous and controlled motivation subscales, and the different perceived competence scales (N of Study 1=230; N of Study 2=228).

Results from correlation analyses		VCCQ^a^study 1	VCCQ study 2
**Convergent validity**			
	TSRQ^b^autonomous motivation subscale	.66^c^	.37^c^
	PCS^d^for reducing alcohol intake	.52^c^	N/A^e^
	PCS for reducing cannabis consumption	N/A	.01(NS)^f^
**Divergent validity**			
	TSRQ Controlled motivation subscale	.29^c^	.37^c^
	PCS for learning	N/A	.05 (NS)

^a^VCCQ: virtual care climate questionnaire.

^b^TSRQ: treatment self-regulation questionnaire.

^c^*P<*.001.

^d^PCS: perceived competence scale.

^e^N/A: not applicable.

^f^NS: nonsignificant.

## Discussion

### Principal Findings

The objective of this study was to develop and validate the VCCQ: the first instrument specifically intended to measure perceived support for autonomy in a virtual care setting. On the basis of on NIRT analyses, we found that several items needed to be excluded from the questionnaire: three items in Study 1 and seven items in Study 2. The final 15-item version included items that were adjusted from the original HCCQ [7] and PASSES [8] items, as well as new Internet-specific items based on expert consultation. This suggests that, as expected, neither the HCCQ nor the PASSES would have sufficiently covered the concept of perceived autonomy-support in a virtual care setting and provide support for the approach that was taken for item development, that is, to use both existing questionnaires and expert consultation as input.

The final 15-item VCCQ was characterized by unidimensionality, monotonicity, and IIO, as required by NIRT. Therefore, the items can be used to compute a total score that reflects the respondents’ relative positions on the latent construct of “perceived autonomy support,” and these scores can also be used for comparing groups. Most items also met the local independence criterion, though before any items can be excluded based on this new psychometric test, further research is needed. Yet the items flagged, based on the criterion of local independence in our studies, can be first candidates for exclusion from a short-form VCCQ. Furthermore, the CFA of the 15-item VCCQ showed an acceptable model fit consistent with MSA results. Fit values were slightly below the recommended thresholds [33,34], though there is a scientific debate on the use of and value that should be attached to these thresholds when determining a scale’s model fit [36]. Reliability of the 15-item VCCQ was confirmed by two alternative tests (alpha and omega). Therefore, this version can be used confidently in future research, while continuing investigations into its measurement properties in different contexts and populations.

All three negatively worded items (ie, 13, 18, and 23) were excluded from the questionnaire due to low inter-item correlations in both studies. While including such items in questionnaires is recommended to reduce acquiescence bias, it may lead to response inconsistencies and may prove difficult to specify in measurement models. Therefore, some experts advise against their use [37,38]. Excluding these items, together with other misfitting items, resulted in good measurement properties of the current VCCQ version without limiting the construct breadth—an item was only excluded when its content showed overlap with other items, it was excluded from the scale in both studies, or was retrospectively deemed inappropriate in the context of Web-based, computer-tailored interventions. Future psychometric work on the VCCQ could investigate the risk of acquiescence in reporting on perceived autonomy support and possible solutions.

The scale’s convergent validity was confirmed by positive associations with autonomous motivation for reducing alcohol intake and cannabis consumption, and with perceived competence for reducing alcohol intake. Divergent validity was confirmed by the nonsignificant association with perceived competence for learning, yet the expected negative or nonsignificant relationship with controlled motivation could not be confirmed. In fact, in both studies we found a significant positive association between perceived support for autonomy and controlled motivation, although weaker than the association with autonomous motivation in Study 1. Moreover, in both our studies, autonomous motivation showed a strong significant positive correlation with controlled motivation (*r*=.68 in Study 1; *r*=.61 in Study 2), supporting our idea that autonomous and controlled motivation may represent different motivational dimensions that can coexist within people. This has also been suggested in earlier SDT-based research into motivational profiles, suggesting not only a high quality (ie, high autonomous and low controlled motivation), but also a high quantity motivational profile (ie, high autonomous and high controlled motivation) that does not necessarily lead to inferior results in terms of learning and physical activity than the high-quality profile [39]. In addition, earlier research has shown that introjected motivation, a form of controlled motivation that is characterized by performance of a behavior to avoid feelings of guilt or anxiety or to attain ego enhancement such as pride [3], may have positive effects on health behavior change [40]. Thus, an increase in especially this type of controlled motivation from using an Web-based intervention may also be a desired outcome. In our studies, perceived support for autonomy as measured by the VCCQ showed a stronger association with the introjected motivation subscale (*r*=.35 in Study 1; *r*=.38 in Study 2) than with the extrinsic motivation subscale (*r*=.21 in Study 1; *r*=.34 in Study 2). Whether this can be the direct result of providing support for autonomy can, however, not be concluded from the present study due to its cross-sectional nature. Following this line of reasoning, an alternative explanation may be that both autonomously motivated and respondents with high levels of controlled motivation were included in the present studies, with both groups finding potentially different elements in the intervention as supportive of their autonomy. Longitudinal research that includes measurement of the different types of motivation both before and after participation in a virtual care intervention is needed to examine whether perceiving higher autonomy support as measured by the VCCQ indeed increases autonomous motivation and reduces or increases the different forms of controlled motivation, and to determine whether autonomy-supportive interventions differ in their effectiveness for people with different types of motivation—in terms of both motivational and behavioral outcome measures. In addition, longitudinal research may be used to obtain evidence related to the questionnaire’s predictive validity.

### Strengths and Limitations

An important strength of this study is that before proceeding to classical test theory analyses to determine the VCCQ’s validity and reliability, we investigated the questionnaire’s structure using NIRT analyses [26]. A known limitation of factor analytic methods is that they do not take into account differences in item difficulty, which are considered in IRT analyses, including MSA [40]. Moreover, as other parametric methods, they attempt to estimate quantitative differences between respondents, and are therefore unnecessarily restrictive for constructs that only refer to differences in degree [26]; perceived autonomy support is arguably among these constructs and more adequately investigated via MSA. So far, not many studies have used NIRT analyses in addition to classical test theory analyses and we are unaware of any previous studies that did so in the context of SDT-based questionnaires.

The VCCQ was developed to measure perceived support for autonomy in a variety of virtual care settings, targeting different kinds of health behavior. Yet, in the present study it was tested for its reliability and validity solely in the context of Web-based interventions aimed at two addictive behaviors, namely alcohol consumption and cannabis use. To increase the generalizability of the results to interventions targeting other nonaddictive health behaviors such as physical activity and healthy dietary behaviors, future research could consider testing the validity and reliability of the VCCQ in the context of other Internet- based interventions. Moreover, as both studies described were cross-sectional, test-retest reliability and predictive validity of the VCCQ could not be ascertained. To investigate these properties, longitudinal research designs should be considered. An additional limitation pertains to the use of a scenario (ie, to ensure that participants made proper use of the Web-based interventions, they were instructed to imagine they wanted to change their behavior and that they used the Web-based intervention to this end). Whereas this may limit the ecological validity of the results presented, the aim of this specific study was to develop and validate a novel questionnaire, and not necessarily to actually help people to improve their health behavior. As a consequence, the exposure of people who truly wanted to change their behavior to interventions that were not necessarily developed to be autonomy-supportive was deemed undesirable. One of the uses of VCCQ is for guiding intervention development, and in such cases our choice of sample is actually recommended: people who are similar enough to the target group to be able to relate to the content, but not require the intervention as such (and thus be in a more vulnerable position). Due to the inclusion criteria set, the participants in the present studies can be considered very well-capable of imagining themselves wanting to change their behavior and of evaluating the interventions in terms of their autonomy-supportiveness. Therefore, the scenario used can be considered rather realistic. Another limitation that warrants attention concerns the use of an odd number of answering categories within the VCCQ, as also used in the HCCQ and PASSES, providing respondents with the possibility to equivocate when completing the questionnaire. In both studies, we prevented this from having a large influence on the psychometric analyses, by excluding respondents with a too high number of neutral answers (ie, respondents who had z-scores >3 for the number of neutral answers), though as is shown by the answering patterns presented in Multimedia Appendix 2 neutral answers were still rather common. The optimal number of response categories for questionnaires assessing support for autonomy in different contexts (virtual or face-to-face) is therefore a valuable research question for further studies. Finally, responding to the final 15 VCCQ-items within multimeasure surveys may still place a significant burden on respondents, potentially increasing nonresponse. Future research may therefore consider the development and validation of a VCCQ-short form, following examples from earlier research where the 15-item HCCQ was transformed into a valid and reliable six-item IOCQ (important other climate questionnaire) [41] and a six-item mHCCQ (modified HCCQ adapted for breast cancer patients) [42].

### Conclusions

By developing the VCCQ, this study aimed to address the lack of validated tools that measure support for autonomy in the context of virtual care settings. Scientifically, the VCCQ enables further research into the role of autonomy-support in virtual care settings without virtual health care providers involved, a context in which evidence for its positive effects is currently lacking. Practically, using the VCCQ will help to identify how Web-based health behavior change interventions can most successfully support autonomy, subsequently increase autonomous forms of motivation, and ultimately promote a healthy lifestyle. Health behavior change interventions are increasingly provided via the Internet [9], and people tend to search for health-related information first and foremost through this medium [10]. While limited resources are available for the widespread implementation of effective lifestyle interventions and health care decision makers should select interventions based on their cost-effectiveness, Web-based health behavior change interventions are likely to be highly competitive when compared with other types of interventions [43,44]. Investigating the role of autonomy support in these settings is necessary, as it may lead to further improvements in the effectiveness of such interventions.

The VCCQ appeared to accurately assess participants’ perceived autonomy-support offered by two Web-based health behavior change interventions. Overall, the scale showed the expected properties and relationships with relevant concepts and the studies presented suggest this first version of the VCCQ to be reasonably valid and reliable. As a result, the current version should be used cautiously in future research and practice to measure perceived support for autonomy within a virtual care climate. However, future research efforts are required that focus on further investigating the VCCQ’s divergent validity, on determining the VCCQ’s validity and reliability when used in the context of Web-based interventions aimed at improving nonaddictive or other health behaviors, and on developing and validating a VCCQ-short form. A Dutch version is available from the authors upon request.

## Appendix

Multimedia Appendix 1 Flow of respondents who started participation.

Multimedia Appendix 2 Answering patterns for all 23 VCCQ (virtual care climate questionnaire) items.

Multimedia Appendix 3 Inter-item correlations.

Multimedia Appendix 4 Item Step Response Functions.

Multimedia Appendix 5 Confirmatory factor analysis diagrams.

## Abbreviations

CFA: confirmatory factor analysis
CFI: comparative fit index
HCCQ: health care climate questionnaire
IIO: invariant item ordering
IOCQ: important other climate questionnaire
IRT: item response theory
ISRF: item step response function
LI: local independence
MSA: Mokken scale analysis
NIRT: nonparametric item response theory
PASSES: perceived autonomy support scale for exercise settings
PCS: perceived competence scale
RMSEA: root mean square error of approximation
SDT: self-determination theory
TLI: Tucker-Lewis index
TSRQ: treatment self-regulation questionnaire
VCCQ: virtual care climate questionnaire
