# Supplementary material for: The Virtual Care Climate Questionnaire: Development and Validation of a Questionnaire Measuring Perceived Support for Autonomy in a Virtual Care Setting
Source: J Med Internet Res. 2017 May 8;19(5):e155. doi: 10.2196/jmir.6714 (PMC5705912; doi:10.2196/jmir.6714)
Supplement: Multimedia Appendix 1 [file jmir_v19i5e155_app1.pdf]

Appendix 1 Flow of respondents who started participation

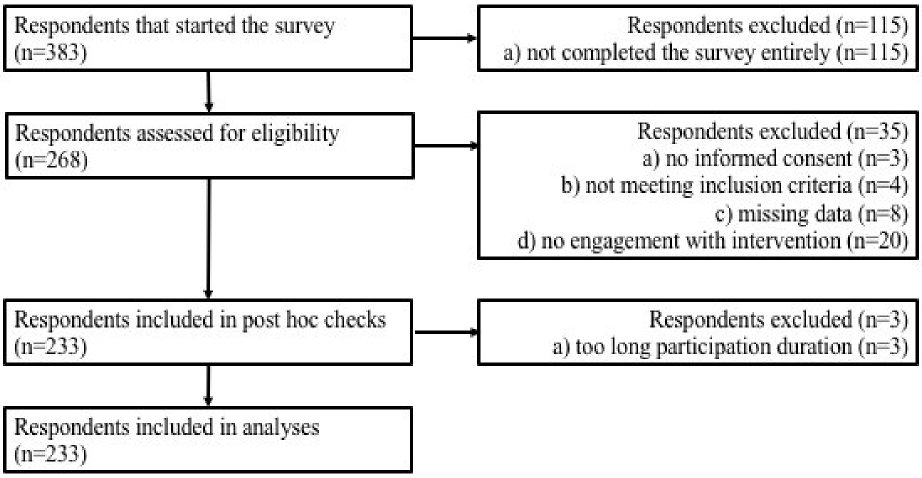

Figure 1.1: *Consort flow-diagram of respondents who started participation in Study 1*

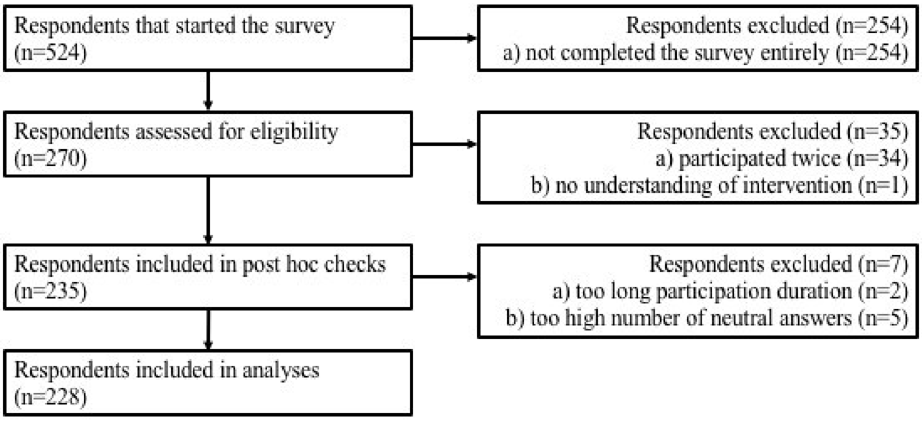

Figure 1.2: *Consort flow-diagram of respondents who started participation in Study 2*
