# Supplementary material for: The Virtual Care Climate Questionnaire: Development and Validation of a Questionnaire Measuring Perceived Support for Autonomy in a Virtual Care Setting
Source: J Med Internet Res. 2017 May 8;19(5):e155. doi: 10.2196/jmir.6714 (PMC5705912; doi:10.2196/jmir.6714)
Supplement: Multimedia Appendix 3 [file jmir_v19i5e155_app3.pdf]

Appendix 3 Inter-item correlations

Table 3.1

Inter-item correlations of the 23 VCCQ items in Study 1 (N=230)

	**1**	**2**	**3**	**4**	**5**	**6**	**7**	**8**	**9**	**10**	**11**	**12**	**13**	**14**	**15**	**16**	**17**	**18**	**19**	**20**	**21**	**22**	**23**
**1**																							
**2**	.76[a]																						
**3**	.56[a]	.60[a]																					
**4**	.60[a]	.60[a]	.46[a]																				
**5**	.71[a]	.73[a]	.67[a]	.65[a]																			
**6**	.65[a]	.68[a]	.47[a]	.72[a]	.68[a]																		
**7**	.60[a]	.66[a]	.45[a]	.66[a]	.60[a]	.76[a]																	
**8**	.69[a]	.73[a]	.59[a]	.66[a]	.72[a]	.72[a]	.69[a]																
**9**	.65[a]	.76[a]	.56[a]	.64[a]	.69[a]	.70[a]	.68[a]	.80[a]															
**10**	.54[a]	.62[a]	.45[a]	.65[a]	.63[a]	.66[a]	.66[a]	.68[a]	.78[a]														
**11**	.55[a]	.58[a]	.48[a]	.67[a]	.64[a]	.69[a]	.64[a]	.66[a]	.69[a]	.76[a]													
**12**	.63[a]	.69[a]	.55[a]	.64[a]	.69[a]	.77[a]	.71[a]	.74[a]	.73[a]	.72[a]	.78[a]												
**13**	-.07	-.09	-.09	.02	-.09	-.06	-.04	-.09	-.08	-.03	-.11	-.06											
**14**	.52[a]	.60[a]	.43[a]	.60[a]	.61[a]	.56[a]	.53[a]	.55[a]	.59[a]	.62[a]	.60[a]	.62[a]	.13[c]										
**15**	.42[a]	.52[a]	.36[a]	.54[a]	.46[a]	.54[a]	.52[a]	.53[a]	.55[a]	.57[a]	.62[a]	.58[a]	.00	.69[a]									
**16**	.55[a]	.55[a]	.42[a]	.66[a]	.54[a]	.62[a]	.65[a]	.58[a]	.55[a]	.58[a]	.59[a]	.63[a]	.00	.62[a]	.66[a]								
**17**	.58[a]	.63[a]	.49[a]	.66[a]	.61[a]	.62[a]	.67[a]	.64[a]	.64[a]	.65[a]	.57[a]	.67[a]	.01	.64[a]	.60[a]	.78[a]							
**18**	.44[a]	.55[a]	.39[a]	.42[a]	.44[a]	.47[a]	.49[a]	.49[a]	.57[a]	.53[a]	.45[a]	.56[a]	.10	.59[a]	.52[a]	.51[a]	.52[a]						
**19**	.62[a]	.49[a]	.67[a]	.67[a]	.62[a]	.69[a]	.62[a]	.63[a]	.64[a]	.61[a]	.62[a]	.64[a]	-.02	.61[a]	.64[a]	.77[a]	.73[a]	.56[a]					
**20**	.64[a]	.64[a]	.52[a]	.68[a]	.66[a]	.70[a]	.69[a]	.66[a]	.66[a]	.62[a]	.68[a]	.69[a]	-.02	.64[a]	.62[a]	.74[a]	.72[a]	.58[a]	.79[a]				
**21**	.61[a]	.56[a]	.56[a]	.59[a]	.64[a]	.61[a]	.60[a]	.58[a]	.59[a]	.52[a]	.56[a]	.62[a]	-.11[d]	.58[a]	.52[a]	.65[a]	.65[a]	.48[a]	.68[a]	.75[a]			
**22**	.64[a]	.71[a]	.64[a]	.57[a]	.73[a]	.63[a]	.62[a]	.70[a]	.72[a]	.58[a]	.57[a]	.69[a]	-.07	.64[a]	.63[a]	.65[a]	.66[a]	.52[a]	.69[a]	.68[a]	.73[a]		
**23**	.15[c]	.18[b]	.03	.27[a]	.11	.16[c]	.20[b]	.16[c]	.22[a]	.28[a]	.20[b]	.17[c]	.15[c]	.23[a]	.30[a]	.25[a]	.23[a]	.32[a]	.33[a]	.21[b]	.08	.16[c]	

Note: Spearman correlation coefficients: [a] $P< .001$, [b] $P< .01$, [c] $P< .05$, [d] $P<.10$.

Table 3.2

Inter-item correlations of the 23 VCCQ items in Study 2 (N=228)

	1	2	3	4	5	6	7	8	9	10	11	12	13	14	15	16	17	18	19	20	21	22	23
**1**																							
**2**	.60[a]																						
**3**	.09	.03																					
**4**	.39[a]	.54[a]	.02																				
**5**	.45[a]	.46[a]	.25[a]	.34[a]																			
**6**	.27[a]	.49[a]	-.05	.48[a]	.39[a]																		
**7**	.38[a]	.54[a]	-.03	.44[a]	.27[a]	.50[a]																	
**8**	.47[a]	.55[a]	.13[c]	.43[a]	.45[a]	.49[a]	.50[a]																
**9**	.33[a]	.49[a]	.16[c]	.33[a]	.48[a]	.49[a]	.45[a]	.57[a]															
**10**	.37[a]	.44[a]	.05	.32[a]	.44[a]	.47[a]	.45[a]	.48[a]	.54[a]														
**11**	.37[a]	.48[a]	.12[d]	.39[a]	.47[a]	.46[a]	.43[a]	.43[a]	.52[a]	.53[a]													
**12**	.36[a]	.54[a]	.07	.34[a]	.51[a]	.45[a]	.43[a]	.49[a]	.47[a]	.52[a]	.64[a]												
**13**	-.22[a]	-.16[c]	-.06	-.09	-.19[b]	-.01	.06	-.07	-.08	-.06	-.03	-.04											
**14**	.41[a]	.53[a]	.13[d]	.40[a]	.42[a]	.45[a]	.47[a]	.51[a]	.52[a]	.45[a]	.56[a]	.59[a]	-.05										
**15**	.24[a]	.36[a]	.06	.21[b]	.27[a]	.31[a]	.26[a]	.17[c]	.34[a]	.34[a]	.46[a]	.37[a]	-.06	.37[a]									
**16**	.32[a]	.47[a]	-.11[d]	.46[a]	.26[a]	.35[a]	.41[a]	.34[a]	.32[a]	.31[a]	.43[a]	.35[a]	-.04	.25[a]	.23[a]								
**17**	.34[a]	.42[a]	.02	.37[a]	.18[b]	.36[a]	.52[a]	.36[a]	.39[a]	.31[a]	.36[a]	.36[a]	.03	.46[a]	.26[a]	.37[a]							
**18**	.14[c]	.06	.05	.12[d]	-.03	.17[b]	.15[c]	.19[b]	.16[c]	.05	.11	.05	.14[c]	.01	.10	.22[a]	.18[b]						
**19**	.36[a]	.51[a]	.05	.43[a]	.35[a]	.53[a]	.46[a]	.46[a]	.49[a]	.49[a]	.46[a]	.45[a]	-.05	.36[a]	.27[a]	.41[a]	.44[a]	.19[b]					
**20**	.44[a]	.48[a]	.06	.42[a]	.44[a]	.49[a]	.45[a]	.48[a]	.50[a]	.48[a]	.43[a]	.43[a]	-.12[d]	.44[a]	.28[a]	.39[a]	.38[a]	.12[d]	.64[a]				
**21**	.33[a]	.26[a]	.22[a]	.37[2]	.32[a]	.29[a]	.32[a]	.31[a]	.37[a]	.28[a]	.37[a]	.23[a]	-.10	.29[a]	.24[b]	.34[a]	.31[a]	.28[a]	.34[a]	.44[a]			
**22**	.36[a]	.49[a]	.12[d]	.34[a]	.41[a]	.37[a]	.38[a]	.43[a]	.42[a]	.49[a]	.43[a]	.50[a]	-.11[d]	.42[a]	.42[a]	.25[a]	.28[a]	.13[d]	.35[a]	.41[a]	.33[a]		
**23**	-.08	-.06	-.03	.04	-.20[b]	.03	.10	-.13[d]	-.10	-.07	-.10	-.11	.41[a]	-.10	-.03	-.03	.14[c]	.28[a]	.02	-.12[d]	-.02	.02	

Note: Spearman correlation coefficients: [a] *P*< .001, [b] *P*< .01, [c] *P*< .05, [d] *P*<.1
