# Supplementary material for: The Virtual Care Climate Questionnaire: Development and Validation of a Questionnaire Measuring Perceived Support for Autonomy in a Virtual Care Setting
Source: J Med Internet Res. 2017 May 8;19(5):e155. doi: 10.2196/jmir.6714 (PMC5705912; doi:10.2196/jmir.6714)
Supplement: Multimedia Appendix 4 [file jmir_v19i5e155_app4.pdf]

Appendix 4 Item Step Response Functions

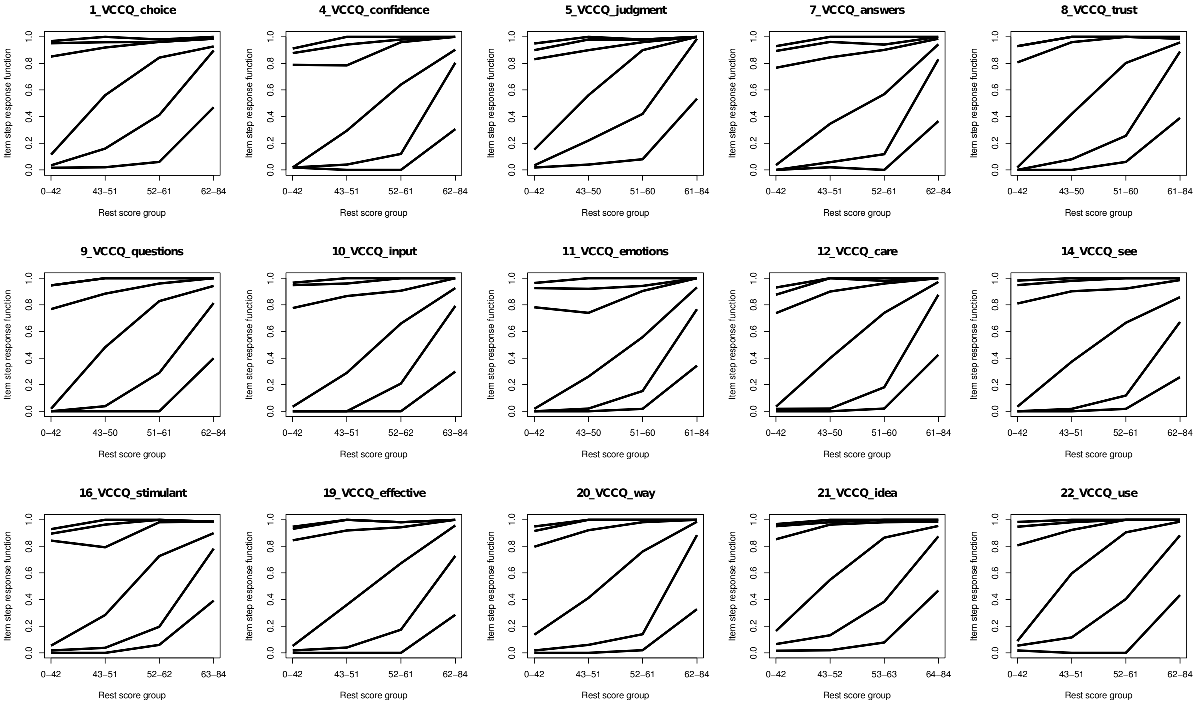

Figure 4.1: *ISRFs for the 15-item VCCQ in Study 1 (N=230)*

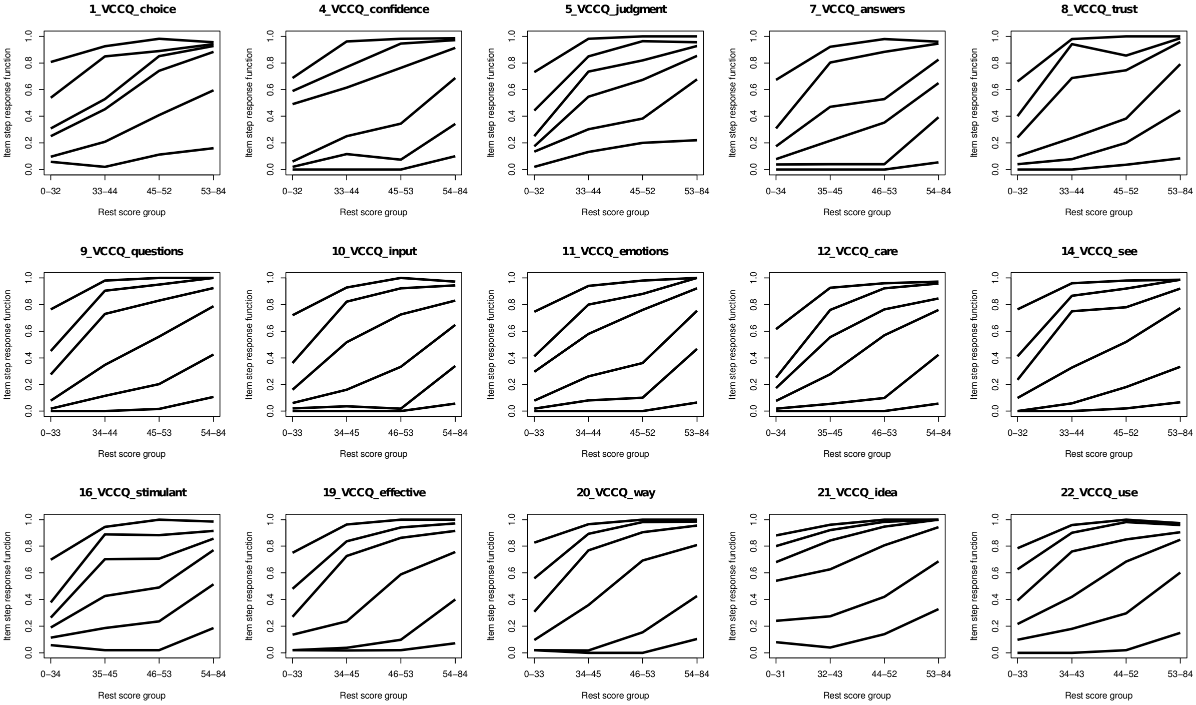

Figure 4.2: *ISRFs for the 15-item VCCQ in Study 2 (N=228)*
