# Supplementary material for: The Virtual Care Climate Questionnaire: Development and Validation of a Questionnaire Measuring Perceived Support for Autonomy in a Virtual Care Setting
Source: J Med Internet Res. 2017 May 8;19(5):e155. doi: 10.2196/jmir.6714 (PMC5705912; doi:10.2196/jmir.6714)
Supplement: Multimedia Appendix 5 [file jmir_v19i5e155_app5.pdf]

**Appendix 5 CFA diagrams**

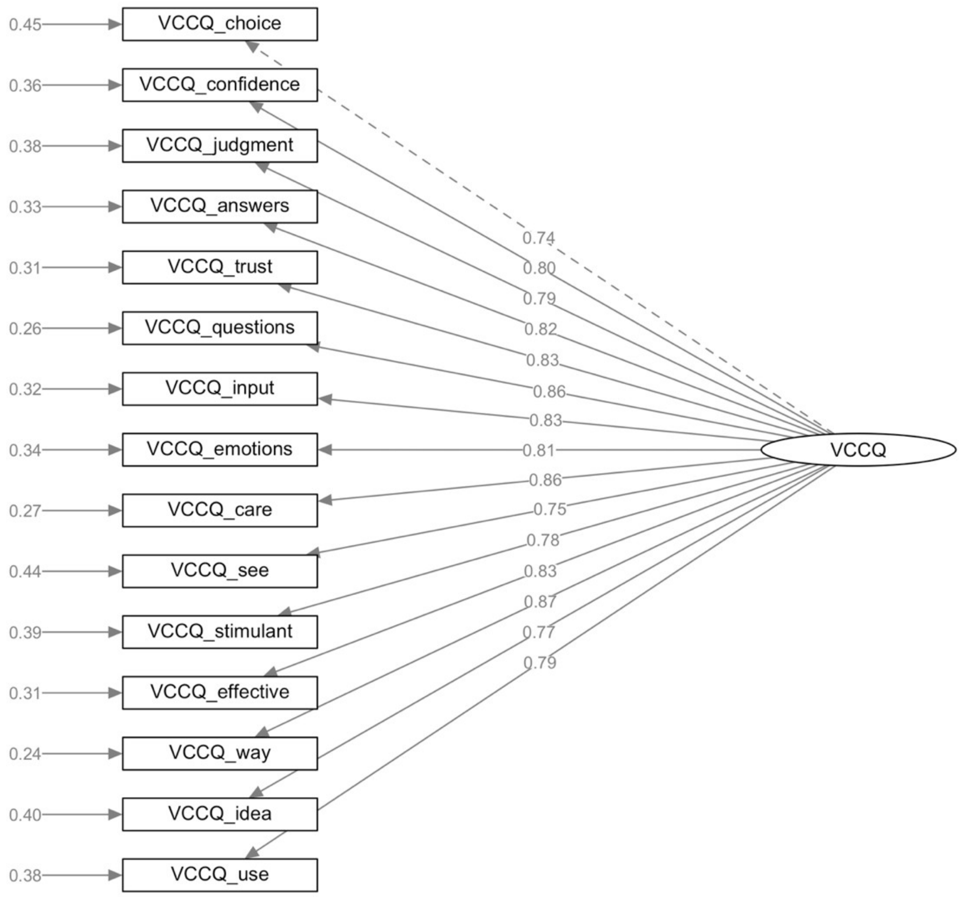


Figure 5.1: *CFA diagram for the 15-item VCCQ in Study 1 (N=230)*

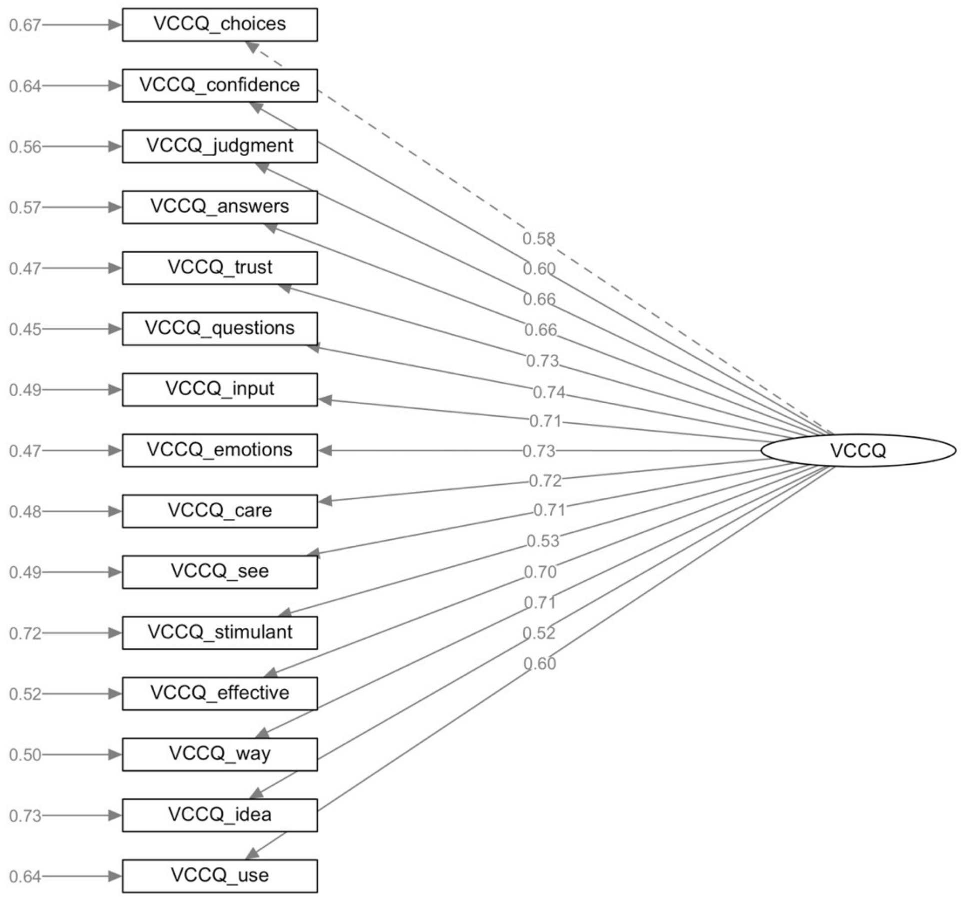


Figure 5.2: *CFA diagram for the 15-item VCCQ in Study 2 (N=228)*
